# Genetic loci associated with tissue-specific resistance to powdery mildew in octoploid strawberry (*Fragaria* × *ananassa*)

**DOI:** 10.3389/fpls.2024.1376061

**Published:** 2024-04-29

**Authors:** Samantha C. Lynn, Jim M. Dunwell, Adam B. Whitehouse, Helen M. Cockerton

**Affiliations:** ^1^ Genetics, Genomics and Breeding, National Institute of Agricultural Botany (NIAB), Kent, United Kingdom; ^2^ Crop Science, University of Reading, Reading, United Kingdom; ^3^ School of Biosciences, University of Kent, Canterbury, United Kingdom

**Keywords:** GWAS, genomic selection, biotroph, plant pathogen, disease resistance

## Abstract

Powdery mildew is one of the most problematic diseases in strawberry production. To date, few commercial strawberry cultivars are deemed to have complete resistance and as such, an extensive spray programme must be implemented to control the pathogen. Here, a large-scale field experiment was used to determine the powdery mildew resistance status of leaf and fruit tissues across a diverse panel of strawberry genotypes. This phenotypic data was used to identify Quantitative Trait Nucleotides (QTN) associated with tissue-specific powdery mildew resistance. In total, six stable QTN were found to be associated with foliar resistance, with one QTN on chromosome 7D associated with a 61% increase in resistance. In contrast to the foliage results, there were no QTN associated with fruit disease resistance and there was a high level of resistance observed on strawberry fruit, with no genetic correlation observed between fruit and foliar symptoms, indicating a tissue-specific response. Beyond the identification of genetic loci, we also demonstrate that genomic selection can lead to rapid gains in foliar resistance across genotypes, with the potential to capture >50% of the genetic foliage resistance present in the population. To date, breeding of robust powdery mildew resistance in strawberry has been impeded by the quantitative nature of natural resistance and a lack of knowledge relating to the genetic control of the trait. These results address this shortfall, through providing the community with a wealth of information that could be utilized for genomic informed breeding, implementation of which could deliver a natural resistance strategy for combatting powdery mildew.

## Introduction

Strawberry powdery mildew is a widespread, ubiquitous disease caused by the fungus *Podosphaera aphanis* (formerly *Sphaerotheca macularis f.* sp. *fragariae*). Uncontrolled epidemics can lead to complete crop abandonment and substantial economic losses for producers (Nellist, 2018). *P. aphanis* is an obligate, biotrophic fungus, from the Erysiphaceae family, which relies on its host for survival. Erysiphaceae spp. infect a wide range of eudicot hosts and, upon establishment, form white powdery mycelia structures, consisting of branched, tubular filaments (hyphae) that cover all above ground plant tissues (leaves, fruit, stolons and flowers) (Kennedy et al., 2013; Takamatsu, 2013). *P. aphanis* undergoes either a prolonged sexual or multiple asexual reproductive cycles, depending upon the season. The fungus overwinters as both mycelium and sexual fruiting structures (chasmothecia) on dormant strawberry plants. The appendages of the chasmothecia intertwine with fungal hyphae anchoring the fruiting body to the surface of the host. In the spring, the ascocarp separates from its host, releasing ascospores that are dispersed via the air current or free water (Gadoury et al., 2010). After landing on a suitable host, the ascospores germinate and penetrate through the plant cell wall before inducing the production of specialised plant-fungal cell structures called haustoria. The fungus then generates aerial conidiophores, which release asexual conidia to enable secondary infection of the host and surrounding plants (Jin and Hall, 2017). Like many powdery mildews, *P. aphanis* has a narrow host range, infecting only strawberry and raspberry plants.

*P. aphanis* undergoes rapid asexual reproduction during the summer and autumn months with optimum temperatures for infection ranging between 15-25°C, where humidity levels are above 75%RH (Amsalem et al., 2006). In infected strawberry leaves, fungal mycelia typically develop first on the underside (abaxial) of the leaf, before spreading to the upper side (adaxial) causing the leaves to curl inwards (Menzel, 2022). Powdery mildew foliage infections lead to a reduction in photosynthesis and thus lower CO_2_ assimilation, and, under severe infections, this can have an indirect impact on yield (Hibberd et al., 1996; Nam et al., 2012; Menzel, 2022). The pathogen also impacts yield directly through infection of strawberry reproductive tissue, causing fruit to become misshapen and restricting or even terminating fruit growth (Menzel, 2022). As a result, powdery mildew outbreaks can lead to 20% to 70% strawberry yield loss annually (Jin and Hall, 2017).

Prevention of powdery mildew epidemics is primarily achieved by the application of chemical pesticides (Berrie and Xu, 2021). However, the number of available actives has been reduced, and restrictions have been placed on the number of fungicide applications allowed per active per year (Berrie and Xu, 2021). Despite these restrictions, *P. aphanis* has evolved resistance to multiple sterol demethylase inhibitors, thus reducing the options available for effective pathogen control (Sombardier et al., 2010). With the emergence of fungicide resistance, there is a greater need to reduce our reliance on fungicide management practices through harnessing natural genetic sources of powdery mildew resistance. Resilient varieties stand to provide an environmentally favourable disease control strategy. Indeed, there is a great need for improved disease resistance levels in elite strawberry germplasm, particularly where everbearing varieties are cropped throughout the growing season leading to prolonged disease exposure. Ultimately, there is a clear requirement for robust and effective disease control strategies as the majority of commercial cultivars still require chemical sprays for powdery mildew control (Berrie and Xu, 2021).

Disease resistance in plants can be either monogenic (controlled by a single genetic element) or polygenic (controlled by multiple genetic elements) (Lindhout, 2002). Major effect genes associated with complete monogenic resistance to powdery mildew have been identified and exploited in breeding programmes across a range of crops (Büschges et al., 1997; Olmstead and Lang, 2002; Zhang et al., 2017). Previous studies have shown that powdery mildew diseases caused by *Podosphaera* spp. infecting hop, melon and apple are controlled by single major resistance genes (Caffier and Laurens, 2005; Cui et al., 2022; Havill et al., 2023). However, no major gene or single locus has been demonstrated to endow complete resistance against *P. aphanis*. In fact, most commercial strawberry cultivars are susceptible to powdery mildew, with only a few strawberry varieties that are deemed to be moderately resistant (Menzel, 2022). Indeed, strawberry powdery mildew resistance characterised to date is highly polygenic (Cockerton et al., 2018; Palmer and Holmes, 2022). Capturing polygenic resistance caused by multiple small-effect alleles may provide a resistance strategy that is more robust than relying on a single gene; however, long-term field experiments are needed to support this theory (Lindhout, 2002; Fondevilla and Rubiales, 2012).

One of the core breeding objectives for temperate strawberry breeding programmes is the development of powdery mildew disease resistant varieties. Understanding the genetic components underlying disease resistance is needed before a genetically informed breeding strategy can be selected to generate highly resistant phenotypes (Palloix et al., 2009; Cockerton et al., 2021). For example, Marker Assisted Breeding can be employed to capture a single major effect resistance gene, such as *Fw1* for resistance to fusarium wilt in strawberry (Soriano et al., 2014; Pincot et al., 2022) or a genomic selection approach can be used to guide the improvement of highly polygenic traits, such as strawberry crown rot resistance (Jiménez et al., 2023). In this study, we seek to gain a greater understanding of powdery mildew resistance and to generate resources that will allow strawberry breeders to capture this polygenic trait. To achieve this, we characterise the genetic elements associated with strawberry powdery mildew resistance through a Genome Wide Association Study and investigate the efficacy of genomic prediction.

## Materials and methods

### Experimental design

A field trial was undertaken to assess powdery mildew disease symptoms across 331 strawberry genotypes. The strawberry association panel included 252 breeding lines and 79 varieties of commercial importance; all material used was adapted for production under temperate north-west European climate. The population contained a mixture of June bearers and everbearers. All stock plant material was housed in a polytunnel before clonal propagation. Five replicate clonal daughter plants were collected per genotype and propagated as misted tips in 9 cm pots containing compost in a heated glasshouse compartment (25°C, 16 hr/8 hr day/night cycle), humidity was set at 100%RH for 2 wks, 80%RH for 2 wks and 60%RH for 2 wks. In August 2020, 1655 strawberry plants were transferred into fumigated polythene raised beds (row length 100 m; space between rows 1 m; spacing between plants 1 m; rows ran from North to South) in an open field at NIAB, East Malling, Kent (51˚17’20.1”N 0˚27’11.0”E); five replicate plants were assessed per genotype. Plants were placed in a randomized block design; the five blocks were arranged along rows and each block contained a single replicate plant per genotype. Trickle irrigation was provided under polythene mulch, and no fungicides were applied to the plot to allow a natural powdery mildew infection to establish.

### Phenotyping

**Figure 1:**
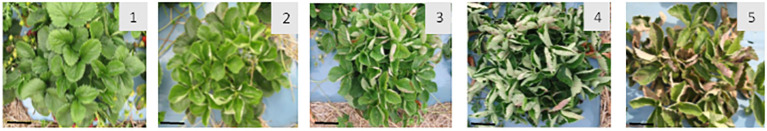
Strawberry foliage powdery mildew disease phenotyping scores: 1. No symptoms, 2. Mild symptoms – upward curling of leaves, 3. Medium symptoms – further upward curling, 4. Severe curling, reddening and leaf damage, 5. Severe necrosis and leaf death 2727. Scale bar 6 cm.

**Figure 2:**
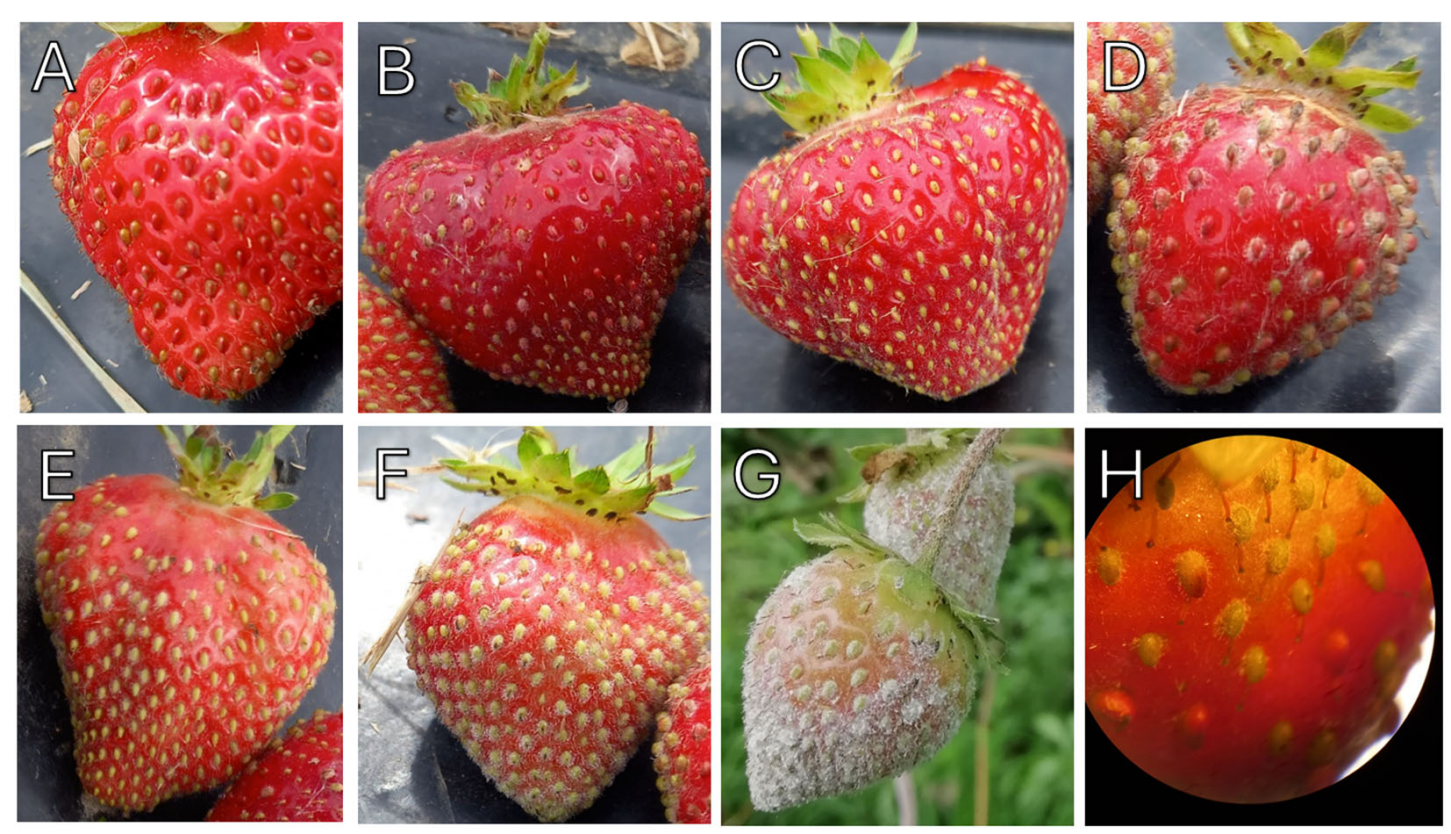
Strawberry fruit powdery mildew phenotyping scores in field samples. **(A)** No mycelium observed on fruit surface **(B)** < 10% of the fruit surface covered with mycelium, **(C)** 10 -25%, **(D)** 25 – 50%, **(E)** 50 – 75% and **(F)** 75 – 100% achenes infected. **(G)** Powdery mildew infection symptoms on strawberry fruit grown under protected production without free water. H. Strawberry achenes on fruit with low powdery mildew presence under the Leica TL3000 Ergo dissection microscope, numerical aperture 0.73-12X.

Foliar disease symptoms were assessed each month from June to October for 2021 and 2022 and scored using a five-point scale (Simpson, 1987). The symptom scoring system was: 1. No symptoms, 2. Slight leaf curling, 3. Leaf curling and mottling, 4. Severe leaf curling, reddening and visible damage to lower leaf surface and 5. Severe necrosis and some leaf death (**Figure 1**). Strawberry fruit powdery mildew infection was assessed in August 2022. Up to five fruits from each plant were scored for disease symptoms. The scoring system was based on a modified protocol from Palmer, 2007; to ensure full visualization of the disease, the fruit was assessed using a x30 jeweller’s loupe. The symptom scoring system was: 0. No superficial mycelium on fruit surface, 1.< 10% of the fruit surface covered with mycelium, 2. 10-25% of the fruit surface covered with mycelium, 3. 25-50% of the fruit surface covered with mycelium, 4. 50-75% of the fruit surface covered with mycelium and 5. 75-100% of the fruit surface covered with mycelium (**Figure 2**).

### Genotyping

Genomic DNA was extracted from newly formed strawberry leaves using the Qiagen DNAeasy plant mini extraction kit (Qiagen Ltd., UK) to the manufacturer’s specifications. Genotyping was performed for 331 accessions using the Affymetrix Istraw90 Axiom array (i90k) (Bassil et al., 2015) or the Istraw 35 384HT Axiom Array (Verma et al., 2017) to generate a total of 22,570 markers; 14,591 of which segregated across the population and were retained in the analysis after filtering. The consensus linkage map denoted 28 groups, with 1 to 7 representing chromosome numbers and A to D representing sub-genome group. Genomic positions of SNPs were defined using the *Fragaria vesca* genome v2.0, with physical positioning of each marker corresponding to a ‘pseudo-octoploid’ chromosome for *Fragaria* × *ananassa*.

### Statistical analysis

Scores for the two replicate years of foliage assessment were analysed independently for 2021 and 2022. The Area Under the Disease Progression Curve (AUDPC) was calculated to combine the foliage disease symptom scoring events. The AUDPC was calculated using the R package ‘agricolae’ (de Mendiburu and de Mendiburu, 2019) as follows:


$$AUDPC=\left\{\sum_{i=1}^{n-1}\left[\frac{y_{i+1}+y_{i}}{2}\right]*[X_{i+1}-X_{i}]\right\}$$

Where *y* is the mildew severity score, for score *i*, *X* represents the time in months and *n* is the number of scoring events. Relative AUDPC (rAUDPC) was calculated by dividing the AUDPC value by the number of phenotyping events. To generate an overall fruit disease score per plant, weighted averages were taken across the five pseudo-replicate fruit disease score assessments. For each phenotype, spatial modelling was used to correct for environmental variation across the field trial. Autospatial correlation analysis was performed in R using the SpaATs package (Rodríguez-Álvarez et al., 2018). Disease scores were corrected for spatial heterogeneity of pathogen incidence across the plot, using penalized splines. Broad sense generalized heritability (H^2^) for genetic associations was calculated using SpATS (Oakey et al., 2006). Best Linear Unbiased Estimates (BLUE) were generated using R package ‘lme4’ through a mixed linear effect model where genotype was specified as a fixed effect and block as a random effect (Bates et al., 2009). The BLUE genotype scores were used for downstream genetic analysis.

### Genetic analysis

A Genome Wide Association Study (GWAS) analysis was conducted using the BLUE foliar disease scores for 2021 and 2022 and BLUE fruit disease scores in 2022 across 331 different genotypes. The GWAS analysis was conducted using PLINK 1.9 association analysis (Purcell et al., 2007; Sobczyk and Harrison, 2023). SNPs were filtered to remove those where the minor allele was represented in less than 5% of the genotypes. Any SNP that was missing in greater than 50% of the population was removed from the analysis. A genomic relationship matrix for the population was produced using the ‘snpready’ R package (**Supplementary Figure S1**; Granato et al., 2018). The analysis was adjusted using principal component co-variates to account for population stratification. A Manhattan plot was produced using the ‘CMplot’ R package to visualize GWAS Bonferroni corrected *p*-value (*p*< 3.423 × 10^–6^) results across the octoploid strawberry chromosomes. The correlation matrices were created using the ‘corrplot’ R package to assess the genotypic and individual Spearman rank correlations between phenotypic fruit and foliage scores.

### Identification of candidate resistance genes

The most significant focal SNP was identified for each QTN. Disease related genes within 100 Kbp of focal SNPs were identified using browser extensible data software (BED tools) (Quinlan and Hall, 2010). Resistance genes identified from the annotated *F. vesca* genome were Nucleotide Binding Site (NBS), Receptor Like Kinase (RLK), Mildew Loci O (MLO), Trans Membrane Coiled-Coiled (TMCC) and Receptor Like Protein (RLP).

### Genomic selection

The potential use of genomic selection in breeding for strawberry powdery mildew resistance was determined. Genomic prediction was performed using the ridge regression best linear unbiased prediction “rrBLUP” R package, or using the R package ‘bwGR’ for Bayes A and Bayes C predictions (Xavier et al., 2020), using the phenotyping data to estimate the effect of each marker on disease score (Endelman, 2011). To achieve this, the population was split into a training sample of 60% and a test sample of 40%. The genotype and phenotype data for the training sample was used to train the model. The test genotyping data was fed into the model to predict the phenotype of the individuals within the test sample. The predicted scores were then correlated with the actual phenotype values to assess the predictive accuracy of the model. The model was run with 100 permutations; for each iteration, genotypes were randomly allocated to either the training or test data set.

## Results

### Foliage disease variance predominantly controlled by genetic factors

**Figure 3:**
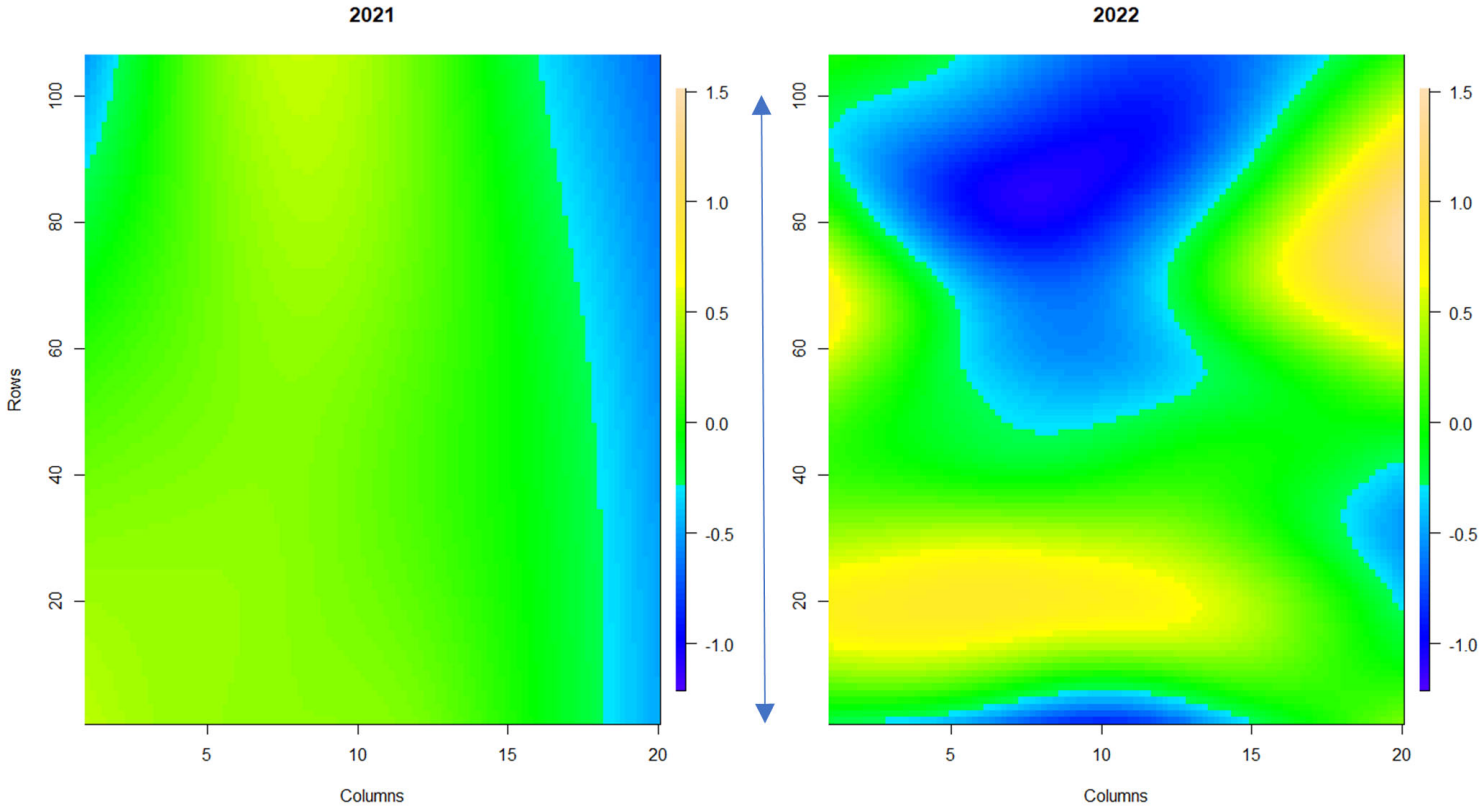
Spatial trends of foliar powdery mildew disease symptoms across strawberry plants in the field plot. The scale bar indicates the relative level of disease incidence from -1.2 (blue, low) to 1.5 (orange, high). Columns denote each raised bed. Rows denote the position of each plant along the raised bed. The blue arrow denotes 100 meters. 1a shows the spatial trend for 2021, 1b shows the spatial trend for 2022.

Powdery mildew disease symptoms were assessed across a replicated field trial of 331 strawberry genotypes in 2021 (assessment of foliage only) and 2022 (assessment of both fruit and foliage). Overall, powdery mildew foliage disease severity was higher in 2022 in comparison to 2021 (**Supplementary Figure S2**), with greater variability in disease symptoms observed across the field plot in 2022 (**Figure 3**). Low levels of infection were observed on the fruit with 67% of genotypes scoring between 0 and 1 (where less than 10% of fruit surface was infected with powdery mildew) (**Supplementary Figure S3**). Broad-sense heritability scores for foliage assessments were 0.83 for 2021 and 0.87 for 2022; indicating that a large proportion of the variation in infection levels was caused by genetic factors. However, powdery mildew fruit infection had a lower broad-sense heritability score of 0.53, indicating a lower influence of genetic factors on disease symptom variation in fruit.

**Figure 4:**
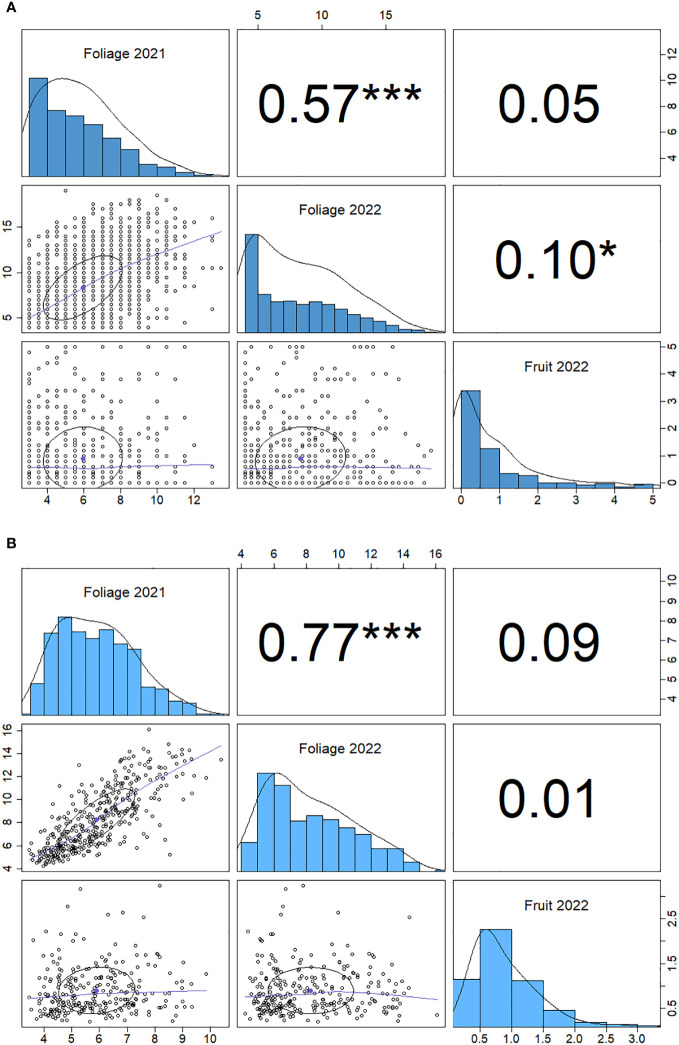
Spearman correlation matrix for powdery mildew phenotype data for foliage phenotypes for 2021 and 2022 and fruit phenotypes 2022. **(A)** genotype correlation and **(B)** individual plant correlation. Significance (*p*) values are denoted by stars: *< 0.05, ***<0.001, numbers are Spearman correlation coefficients (r values).

There is a positive correlation between 2021 and 2022 foliage disease phenotypes (*p*<0.001) for both the genotypic and individual correlations (**Figure 4**). The foliage and fruit infection levels from 2022 showed a weak positive correlation (*p*<0.05) when paired measurements were taken from the same plants. By contrast, the fruit and foliage disease scores for 2022 did not demonstrate a significant genotypic correlation.

### Multiple stable and transient QTN associated with foliar resistance to strawberry powdery mildew

**Figure 5:**
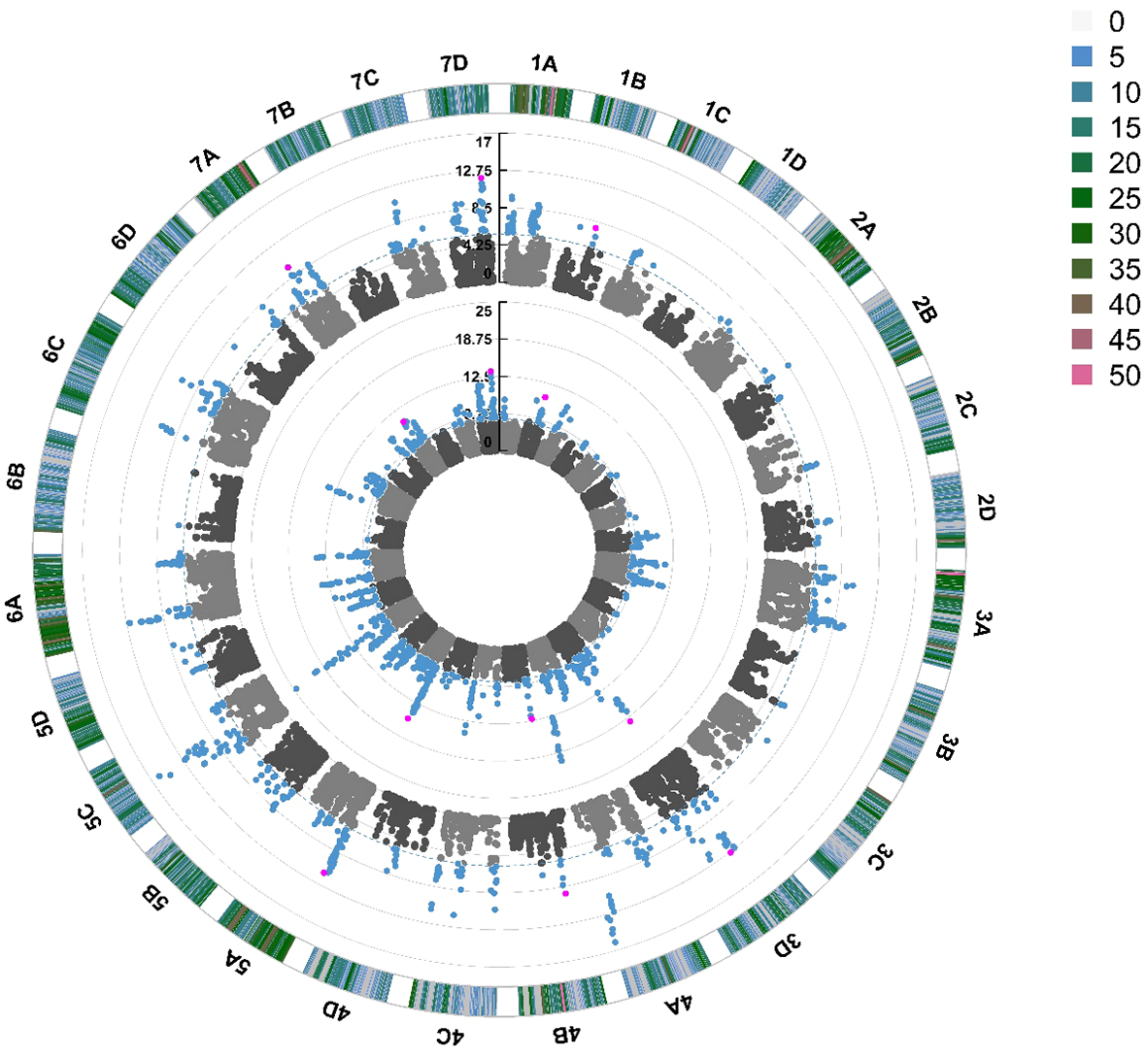
Manhattan plot of SNP markers across the 28 linkage groups of *Fragaria × ananassa* illustrating the relative association of SNPs with powdery mildew foliar disease symptom expression. Points represent markers. Blue points represent markers that fall above the -log^10^(*p*) significance threshold represented by the blue dotted line. Pink points represent stable QTN identified in 2021 and 2022. The inner circle represents SNPs associated with foliar powdery mildew disease symptoms in 2022, middle circle in 2021. The outer circle represents the density of SNPs present on each chromosome within a 1 Mb window, with reference to the key coded from 0 to >49 SNPs.

Quantitative Trait Nucleotides (QTN) were associated with foliar powdery mildew disease resistance on 19 out of 28 chromosomes in 2021 and 2022 (**Figure 5**). The same QTN were identified across both years on chromosomes 3D, 4A, 5A, 5C, 6A and 7D. The most significant stable QTN was *FaRPa7Dab* an additive allele, present on Chromosome 7D with two copies of the resistance allele associated with a 61% effect on resistance. There were two different QTN observed on chromosome 6B across 2021 and 2022; these were only 100 kbp apart (6.5 Mb and 6.6 Mb) and thus these QTN neighbour the same cluster of resistance genes. However, as one QTN behaves in a dominant fashion and the other in an additive fashion, it is clear that these QTN represent two discrete transient alleles present in the same genetic region. Multiple QTN were identified as significantly associated with foliage disease resistance in 2021 and 2022; by contrast, no significant QTN were associated with fruit disease resistance (**Supplementary Figure S4**).

For each QTN, resistance genes were identified within 100 kbp of the focal SNPs (**Table 1**). GWAS analysis led to the identification of six stable QTN (*FaRPa1Bab, FaRPa3Dab, FaRPa4Bab, FaRPa5Aab, FaRPa7Aab and FaRPa7Dab*) associated with powdery mildew resistance over both years. The focal SNP representing *FaRPa3Dab* was associated with both Receptor Like Kinase (RLK) and Receptor Like Protein (RLP) resistance genes and the focal SNP representing *FaRPa7Dab* was associated with a RLK and Nuclear Binding Site (NBS) resistance gene. *FaRPa5Aab* was associated with RLK and TransMembrane Coiled Coil (TMCC) resistance genes. The *FaRPa3Dab* QTN is located directly inside a putative disease RLP resistance gene (mrna03571.1-v1.0-hybrid), which functions as a pattern recognition receptor (PRR) and recognises the presence of pathogens and initiates Pathogen Activated Molecular Pattern (PAMP) triggered immunity. *FaRPa7Dab* was associated with RLK, TM (transmembrane) domains with C terminal Leucine Rich Repeat (LRR) and NBS, TIR Toll/Interleukin-1 (TIR) genes. One gene associated with *FaRPa7Dab* was identified as a putative plant disease resistance gene, homologous to a TMV resistant protein that contains a TIR domain and P-loop containing nucleoside triphosphate hydrolase, which is involved in signal transduction and disease and stress response.

**Table 1:** Focal Single Nucleotide Polymorphisms (SNP) representing Quantitative Trait Nucleotides (QTN) associated with strawberry powdery mildew resistance after GWAS analysis for 2021 and 2022 foliar assessment and effect size for each focal SNP with linkage group and position on octoploid consensus map scaled to *Fragaria vesca* genome v2.0. Gene No. indicates the number of resistant genes within 100 kb of the focal SNP.

QTN Name	Linkage group	Position (Mb)	Focal SNP	Type of Gene(s)	Effect size 2021 (%)	Effect size 2022 (%)	Gene No.	Model	Alleles
*FaRPa1Bab*	1B	14.8	**Affx-88817415**	NBS, TMCC	20.2	26.7	2	Additive	3
*FaRPa3Dab*	3D	14.6	**Affx-88838088**	RLK, RLP	**35.8**	**48.9**	2	No minor hom	2
*FaRPa4Bab*	4B	7.7	**Affx-88848257**	TMCC	**38.2**	**38.2**	1	Additive	3
*FaRPa5Aab*	5A	2.3	**Affx-88859881**	TMCC, RLK	25.5	**31.9**	3	Additive	3
*FaRPa7Aab*	7A	12.9	**Affx-88892535**	RLP, TMCC	24.1	28	2	Additive	3
*FaRPa7Dab*	7D	19.8	**Affx-88899847**	RLK, NBS	**36.9**	**60.5**	5	Additive	3

Type of gene illustrates the resistance gene identified as flanking the QTN: Receptor Like Kinase (RLK), Receptor Like Protein (RLP), TransMembrane Coiled Coil (TMCC), Mildew Locus O (MLO), Nuclear Binding Site (NBS). Effect size indicates the change in magnitude of powdery mildew symptoms associated with the QTN. Alleles indicates the number of genotype combinations present in the population. Model represents genetic control of alleles in the presence of powdery mildew, no minor homozygote (no minor hom). Bold effect shows focal SNP representing stable QTN identified in both 2021 and 2022 and effect sizes over 35%.

In addition to the stable QTN, twenty-six transient QTN were identified, in either 2021 or 2022. These included the focal SNP Affx-88876085 representing *FaRPa6Cb*, an additive transient QTN located on chromosome 6C, with two copies of the resistance allele associated with a 78.7% increase in resistance in 2022. The resistance genes neighbouring this transient QTN encode RLK and RLP proteins.

**Figure 6:**
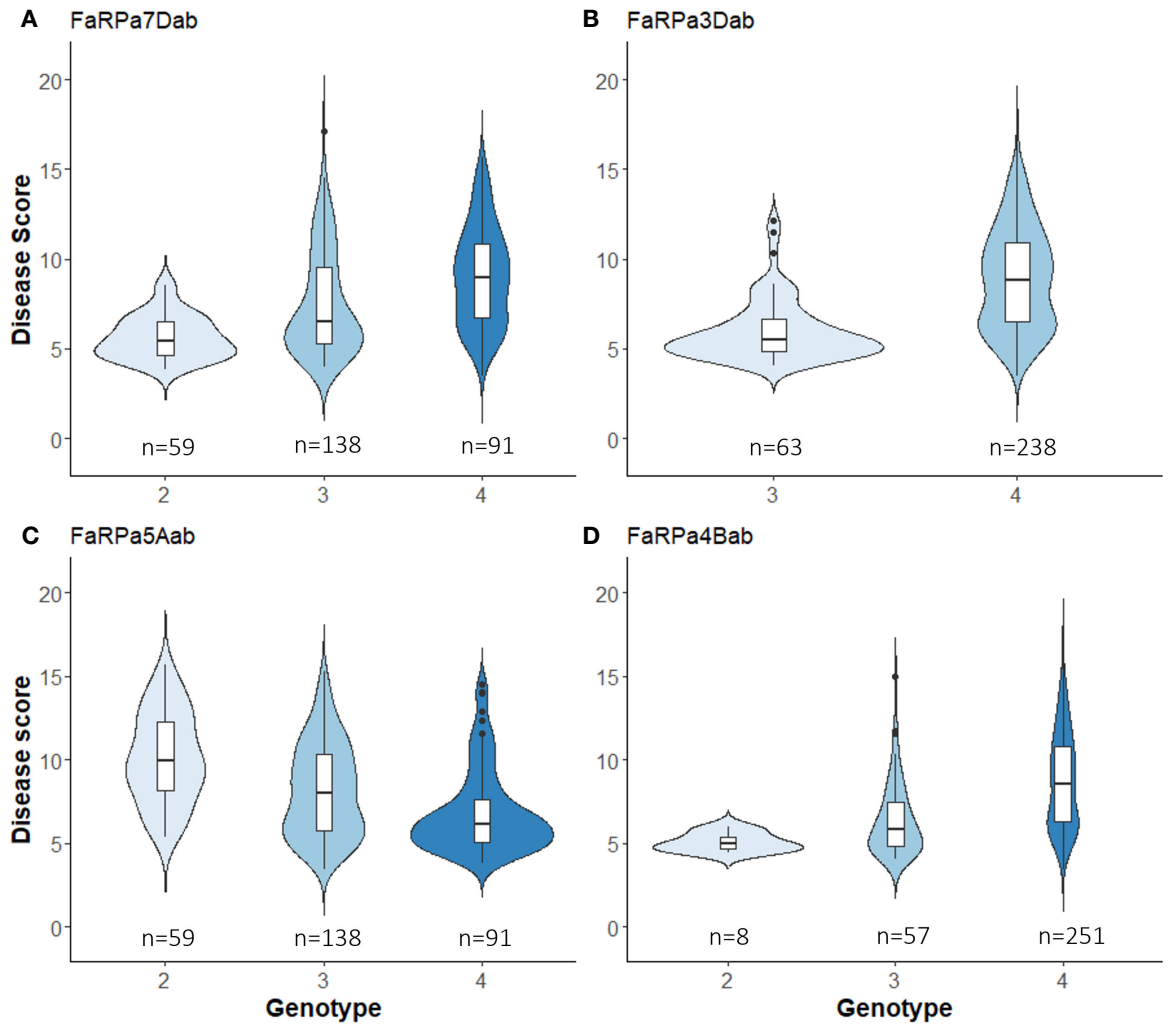
Boxplot of powdery mildew Best Unbiased Linear Estimate foliage disease score for each genotype of stable QTN associated with a large effect size. **(A)**, *FaRPa7Dab* on chromosome 7D (*F*_2,314 _= 27.89, *p*<0.0001), **(B)** *FaRPa3Dab* on chromosome 3D (*F*_1,299 _= 60.26, *p*<0.0001), **(C)** *FaRPa5Aab* on chromosome 5A (*F*_2,285 _= 25.79, *p*<0.0001) and **(D)** *FaRPa4Bab* on chromosome 4B (*F*_2,313 _= 21.23, *p*<0.0001).

In 2022, the stable QTN *FaRPa7Dab* was associated with an effect size of 61% where two copies of the resistance allele were present, with *FaRPa3Dab*, *FaRPa4Bab* and *FaRPa5Aab* associated with effect sizes of 49%, 38% and 32%, respectively. All four of the stable QTN had high effect sizes and were identified in both foliage phenotyping events. No minor homozygote was present for *FaRPa3Dab*; therefore, no information could be gathered on the inheritance for this genetic component (**Figure 6**).

**Figure 7:**
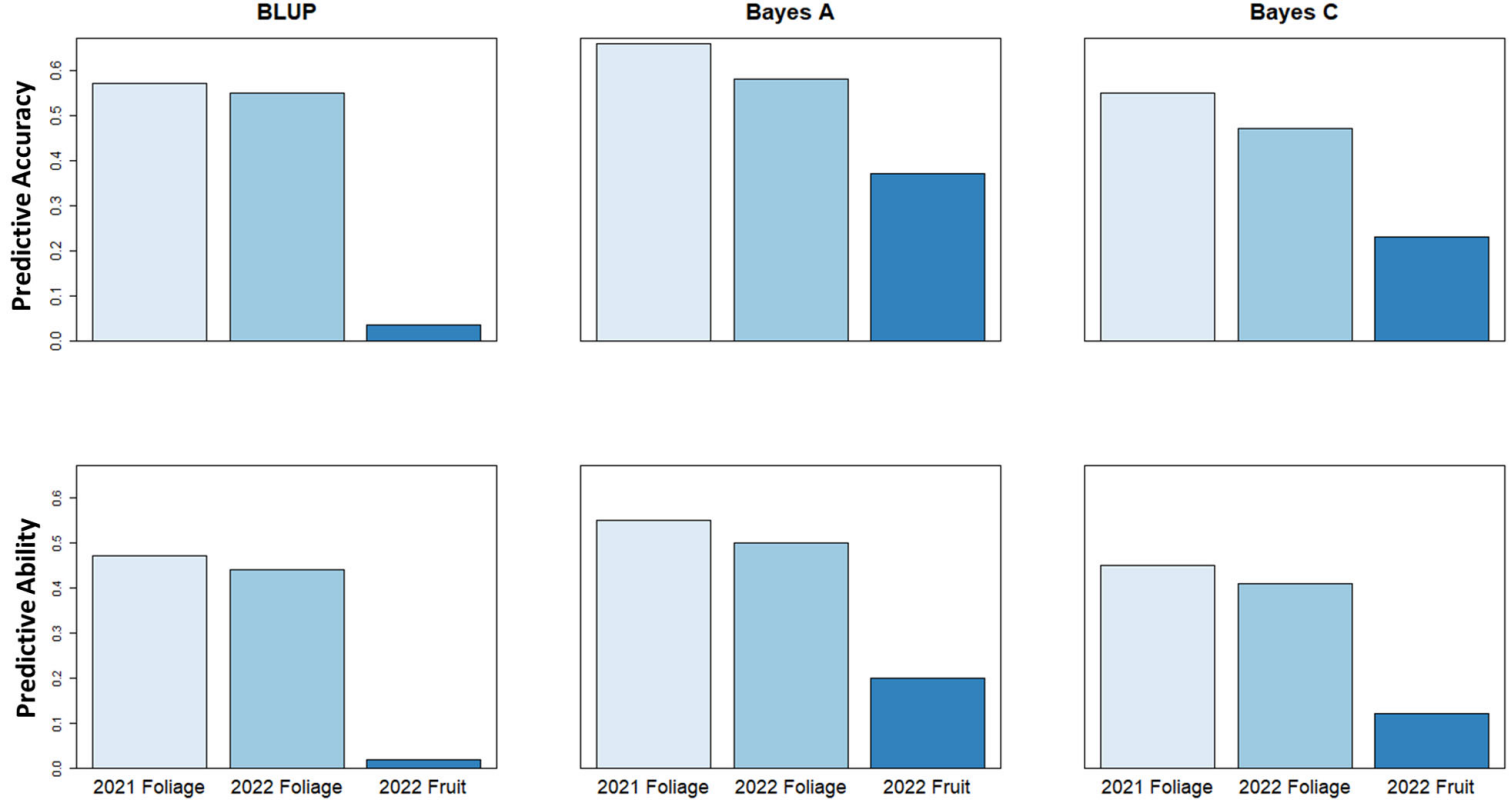
Genomic Selection predictive accuracy and predictive ability values using BLUP, Bayes A & Bayes C genomic selection models to predict strawberry powdery mildew disease severity.

Genomic selection analysis for the 2021 and 2022 foliage disease phenotypes indicated predictive accuracy values of 0.66 and 0.58, and predictive ability of 0.55 and 0.5, respectively using a Bayes A model. However, the predictive accuracy score associated with fruit disease phenotypes was lower at only 0.37, with a predictive ability of 0.20 using a Bayes A model (**Figure 7**). These values show the relative potential of increasing cultivar resistance through genomic selection in the study population.

## Discussion

Most commercial strawberry varieties are susceptible to powdery mildew infection (Menzel, 2022). Efforts to improve resistance are complicated by the fact that this trait is complex and typically controlled in a polygenic fashion, rather than through major effect genes (Cockerton et al., 2018; Sargent et al., 2019; Tapia et al., 2021). A greater understanding of the genetic factors influencing the trait is required to enhance resistance through genomic informed breeding strategies. Here we address the shortage of information, through using a GWAS to identify allelic variants associated with powdery mildew resistance across a range of diverse germplasm. A large number of genetic alleles were identified that were associated with foliar disease resistance. Critically, using a GWAS approach allowed the identification of genetic markers that have linkage with the causative alleles across the wider germplasm. This benefit is not typically shared by the markers identified in Quantitative Trait Loci studies (which artificially generate linkage between markers and the causal genetic element). Ultimately, this retained linkage means that the alleles identified using a GWAS are directly useful for molecular assisted plant breeding (Hamblin et al., 2011). Moreover, the high level of resistance associated with some of the stable QTN, means that this data contains a valuable set of markers, which could be exploited for the generation of disease resistant varieties.

In total, six stable QTN were associated with foliar disease resistance across both years and thus provided universal protection against strawberry powdery mildew. Two of these stable QTN displayed a substantial effect on resistance, and the remaining QTN were associated with effects sizes above 20%. As reported by other researchers, the QTN identified in this study were associated with partial resistance to powdery mildew (Cockerton et al., 2018; Sargent et al., 2019; Feng et al., 2020). Therefore, our findings support the suggestion that, stacking of multiple resistance alleles through Marker Assisted Breeding or Genomic Prediction, could prove desirable breeding approaches to develop high disease resistance strawberry cultivars.

Several QTN were only identified in one of the assessment years. In 2021, thirteen transient QTN were associated with foliar disease resistance and an additional thirteen different transient QTN were identified in 2022. A similar pattern of stable and transient genetic loci has been observed in biparental studies (Cockerton et al., 2018). The observation of transient QTN supports the hypothesis that resistance genes may have an environmental and/or race specific response to powdery mildew infection (Davik and Honne, 2005; Asalf et al., 2013; Carisse et al., 2013). The transient QTN *FaRPa6Db* was found in the 2022 analysis at a location that corresponds to a powdery mildew QTL identified in a bi-parental investigation (Cockerton et al., 2018). The exact same genetic marker was identified (Affx-88904022) at 38.9 Mb on chromosome 6D with three neighbouring RLK resistance genes. This resistance QTL was identified in the “Red Gauntlet” cultivar in four separate phenotyping events (Cockerton et al., 2018). As such, it is evident that this locus plays a role in strawberry powdery mildew resistance and the function of the resistance genes in this region should be investigated further.

The QTN associated with powdery mildew disease resistance were in close proximity to a variety of putative disease resistance genes. Many of the resistance genes identified in this study were cell surface receptors (RLK and RLP’s), which detect the presence of the pathogen and activate the plants immune response. These receptor genes are known to play a role in powdery mildew resistance; indeed, an RLK has been identified as responsible for non-host complete resistance in barley to the wheat adapted form of powdery mildew (*Blumeria graminis f.sp. tritici*) (Rajaraman et al., 2016). Furthermore, several resistance genes containing NBS-LRR domains were identified in this study. NBS-LRR proteins are intracellular immune receptors that can lead to plant cell death through the hypersensitive response; these receptors act as an “on/off” switch and negatively regulate resistance through degradation in response to pathogen effector detection (Dangl and Jones, 2001; Belkhadir et al., 2004). NBS-LRR have been found to provide protection against powdery mildew in grape vine, common bean, and wheat (Goyal et al., 2020; Binagwa et al., 2021; Wang et al., 2022). As multiple candidate resistance genes have been detected in this study, future work should look to determine the function of these candidate genes and ultimately stack validated resistance genes into a single cultivar. This strategy may prove more successful than a single gene strategy, particularly when combining resistance genes representing different pathogen defence mechanisms, as this has been shown to provide more robust resistance and increase the longevity of protection against infection (Dangl and Jones, 2001).

*Mildew Locus 0* (*MLO*) genes are highly desirable breeding targets as they act as susceptibility loci required for host recognition; thus, two copies of a loss-of-function *MLO* allele provide a source of complete and robust resistance (Jørgensen, 1992; Büschges et al., 1997). An *MLO* susceptibility gene was associated with the QTN *FaRPa3Bb* identified on chromosome 3B. This *MLO* gene (mrna31264.1-v1.0-hybrid), corresponds to the *FvMLO16* gene found to be associated with powdery mildew resistance in *F. vesca* (Jambagi and Dunwell, 2017) and Verticillium resistance in octoploid strawberry (Cockerton et al., 2019) and with homologues of the *MLO* associated with powdery mildew resistance in apple (Pessina et al., 2014).

The mildew resistance of foliage was shown to be under strong genetic control with high broad-sense heritability values of over 83% for both years of assessment. These findings correspond with those of Nelson et al. (1995) but were higher than reported by Tapia et al. (2021) and Davik and Honne (2005) who observed a moderate level of heritability (Nelson et al., 1995; Davik and Honne, 2005; Tapia et al., 2021). Heritability values depend upon the variation that is present within the study material; as such, it is clear that our study population contains a relatively large proportion of genetically controlled variation, which can be selected upon by a breeder. A strong genetic correlation was observed between foliage disease scores in 2021 and 2022, adding to the evidence that there was a strong genetic component controlling powdery mildew disease resistance in foliage. Any variation in cultivar disease resistance over the two years could be accounted for, either by variation in the level of disease pressure, variation in the race of the pathogen infecting (resulting from the natural source of inoculum) or the environmental conditions, specifically considering an unprecedented heat wave, reaching 38°C, in August 2022 (Nelson et al., 1996; Blanco et al., 2004; Asalf et al., 2013; Carisse et al., 2013). In contrast to foliage results, strawberry fruit powdery mildew resistance heritability was moderate, with 53% of observed variation accounted for by genetic components. Overall, there was a low incidence of powdery mildew observed on the strawberry fruit, despite the prevalence of powdery mildew on the foliage in 2022. This low level of phenotypic variation present within the population may explain, in part, the lack of ability to discern genetic regions associated with the trait.

A genomic prediction model was used to calculate the predictive ability to improve strawberry powdery mildew resistance. Our results indicate the large potential of genomic selection as a tool to increase foliar mildew resistance, with the ability to capture over 50% of the observed variation in the disease. This value is greater than a predictive ability of 38% seen in similar studies using six full-sib families as a training population (Tapia et al., 2021). By contrast, genomic prediction should not be used to improve fruit powdery mildew resistance, at least not when using the material within, or related to the study population.

Foliage disease phenotypes were not genetically correlated with fruit disease phenotypes, suggesting that two different genetic mechanisms may control disease resistance in the leaves and fruit. Differences were also observed in heritability between foliage and fruit. Furthermore, there were abundant QTN associated with foliage resistance and no QTN associated with fruit resistance. When these findings are combined, it leads to the hypothesis that strawberry powdery mildew resistance is tissue specific. Future work should look to discern the mechanism of tissue specific disease resistance in order to enable selection for both fruit and foliage resistance in breeding programmes. However, given the prevalence of the pathogen, the low fruit disease symptoms indicates that high fruit resistance was present across the germplasm; therefore, introducing durable foliage resistance alone could be sufficient to minimise inoculum and minimise the impact of this pathogen (Menzel, 2022).

In this study, tissue specific genetic loci were associated with foliar-specific resistance but not fruit-specific resistance to strawberry powdery mildew. Tissue specific expression of TGA transcription factors has been observed in strawberry leaves, stems and roots (fruit was not investigated), of these TGA’s, six were also differentially expressed during foliar powdery mildew infection (Feng et al., 2020). Further experiments could investigate the interplay between tissue specific and disease specific expression of TGA. Genetic elements associated with tissue-specific powdery mildew resistance have also been identified in other crops. Multiple tissue-specific QTL were observed to protect grape vine stems (but not fruit or leaves) from powdery mildew infection, alongside a single universal (tissue independent) resistance gene that was effective across observed tissues (Karn et al., 2021). Furthermore, tissue specific resistance has been observed in response to wheat powdery mildew when a resistance gene was introgressed from a wild relative. After transfer, the gene was seen to endow tissue specific resistance on leaves and stems; however, the wheat spikelets remained susceptible to the disease (Zhang et al., 2016).

Understanding the genetic components involved in disease resistance is an important part of informing genetic guided improvement to achieve resilient cultivars (Palloix et al., 2009). Strawberry powdery mildew resistance breeding is a complicated pursuit, as natural resistance is typically incomplete and polygenic in nature. However, this complication may also come with the benefit that capture and exploitation of polygenic resistance, may have greater durability in the field (Davik and Honne, 2005; Palloix et al., 2009).

## Conclusion

We have identified multiple large- to- moderate effect genetic loci that contribute to strawberry powdery mildew disease resistance. Most importantly, the association between the identified markers and the causative alleles is maintained across the population. As such, this data will allow marker assisted breeding to be incorporated into strawberry breeding programmes to develop elite varieties with durable disease resistance. Moreover, we have confirmed that a Bayes A genomic selection approach can be used to capture over 58% of the genetic variation associated with foliage resistance present in the population. As there was no genetic correlation between fruit and foliar symptoms and there were no QTN associated with fruit disease resistance, our results lead us to hypothesise that fruit and foliage mildew resistance is mediated by two different genetic mechanisms and that strawberry powdery mildew resistance is tissue specific.

## Data availability statement

The raw data supporting the conclusions of this article will be made available by the authors, without undue reservation.

## Author contributions

SL: Conceptualization, Data curation, Formal Analysis, Investigation, Methodology, Validation, Visualization, Writing – original draft. JD: Supervision, Writing – review & editing. AW: Resources, Writing – review & editing. HC: Conceptualization, Funding acquisition, Supervision, Writing – review & editing.

## Supplementary material

Supplementary Figure 1 Genetic relationship matrix across 331 strawberry genotypes individuals used in the Genome Wide Association Study to detect powdery mildew resistance genes.

Supplementary Figure 2 Frequency histogram to show powdery mildew foliage disease scores in 2021 (Blue) and 2022 (Pink) for each genotype. Overlapping values (Purple) Values are Best Linear Unbiased Estimates for each genotype calculated from the Area Under the Disease Progression Curve scores that have been corrected for spatial variation.

Supplementary Figure 3 Frequency histogram to show powdery mildew fruit disease scores in 2022 for each genotype. Values are Best Linear Unbiased Estimates for each genotype calculated from the weighted averages across pseudo-replicate berries that have been corrected for spatial variation.

Supplementary Figure 4 A Manhattan plot of SNP markers across the 28 linkage groups of *Fragaria × ananassa* illustrating the relative association of SNPs with powdery mildew fruit disease symptom expression. Points represent markers. Blue points represent markers that fall above the-log10(*p*) significance threshold represented by the black dotted line. The inner circle represents the SNP associations with fruit powdery mildew disease symptoms. The outer circle represents the density of SNPs present on each chromosome within a 1 Mb window, the key represents the number of SNPs segregating from 0 to >49.

Supplementary Table 1 Significant focal Single Nucleotide Polymorphisms (SNPs) associated with strawberry powdery mildew resistance after GWAS analysis for 2021 and 2022 foliar assessment and effect size for each focal SNP. Quantitative trait nucleotide (QTN) name with linkage group and position found. Gene No. indicates the number of resistant genes within 100 kb of the focal SNP. Type of gene illustrates the resistance gene identification by flanking molecular marker: Receptor Like Kinase (RLK), Receptor Like Protein (RLP), TransMembrane coiled coil (TMCC), Mildew loci O (MLO), Nuclear Binding Site (NBS). Effect size indicates the change in magnitude of powdery mildew symptoms associated with the QTN. Alleles indicates the number of genotype combinations present in the population. Model represents genetic control of alleles in presence of powdery mildew, no minor homozygote (no minor hom). Bold effect shows focal SNPs representing stable QTN identified in both 2021 and 2022 and effect sizes over 35%.
