# Supplementary material for: Genetic loci associated with tissue-specific resistance to powdery mildew in octoploid strawberry (Fragaria × ananassa)
Source: Front Plant Sci. 2024 Apr 29;15:1376061. doi: 10.3389/fpls.2024.1376061 (PMC11089197; doi:10.3389/fpls.2024.1376061)
Supplement: Supplementary Figure 1 — Genetic relationship matrix across 331 strawberry genotypes individuals used in the Genome Wide Association Study to detect powdery mildew resistance genes. [file DataSheet_1.docx]

**Supplementary Table 1 & Figures 1-4**

**Supplementary Table 1:** Significant focal Single Nucleotide Polymorphisms (SNPs) associated with strawberry powdery mildew resistance after GWAS analysis for 2021 and 2022 foliar assessment and effect size for each focal SNP. Quantitative trait nucleotide (QTN) name with linkage group and position found. Gene No. indicates the number of resistant genes within 100 kb of the focal SNP. Type of gene illustrates the resistance gene identification by flanking molecular marker: Receptor Like Kinase (RLK), Receptor Like Protein (RLP), TransMembrane coiled coil (TMCC), Mildew loci O (MLO), Nuclear Binding Site (NBS). Effect size indicates the change in magnitude of powdery mildew symptoms associated with the QTN. Alleles indicates the number of genotype combinations present in the population. Model represents genetic control of alleles in presence of powdery mildew, no minor homozygote (no minor hom). Bold effect shows focal SNPs representing stable QTN identified in both 2021 and 2022 and effect sizes over 35%.

| 2021 |  |  |  |  |  |  |  |  |
| --- | --- | --- | --- | --- | --- | --- | --- | --- |
| **QTN Name** | **Linkage group** | **Position (Mb)** | **Focal SNP** | **Type of Gene** | **Effect size** | **Gene No** | **Model** | **Alleles** |
| *FaRPa1Aa* | 1A | 2.0 | Affx-88810185 | RLP, RLK | 17.2 | 2 | Dominant | 3 |
| *FaRPa1Bab* | 1B | 14.8 | **Affx-88817415** | NBS, TMCC | 20.2 | 2 | Additive | 3 |
| *FaRPa2Da* | 2D | 14.5 | Affx-88822125 | TMCC | 16.9 | 1 | Additive | 3 |
| *FaRPa3Aa* | 3A | 10.0 | Affx-88843277 | RLK | 16.2 | 1 | no minor hom | 2 |
| *FaRPa3Ca* | 3C | 8.4 | Affx-88835462 | RLK | 13.8 | 3 | Dominant | 3 |
| *FaRPa3Dab* | 3D | 14.6 | **Affx-88838088** | RLK, RLP | **35.8** | 2 | no minor hom | 2 |
| *FaRPa4Bab* | 4B | 7.7 | **Affx-88848257** | TMCC | **38.2** | 1 | Additive | 3 |
| *FaRPa4Da* | 4D | 16.3 | Affx-88853237 | RLK | 11.4 | 1 | Additive | 3 |
| *FaRPa5Aab* | 5A | 2.3 | **Affx-88859881** | TMCC, RLK | 25.5 | 3 | Additive | 3 |
| *FaRPa5Ba* | 5B | 3.3 | Affx-88860439 | RLK, RLP | 15.7 | 2 | Additive | 2 |
| *FaRPa5Ca* | 5C | 12.8 | Affx-88865131 | TMCC | **39.2** | 1 | Additive | 3 |
| *FaRPa6Aa* | 6A | 6.6 | Affx-88876363 | RLK, RLP | **36.6** | 4 | Additive | 3 |
| *FaRPa6Ba* | 6B | 6.5 | Affx-88876423 | TMCC, RLK, RLP | 17.8 | 5 | Additive | 3 |
| *FaRPa6Ca* | 6C | 32.0 | Affx-88880233 | RLK, TMCC | 21.3 | 2 | Additive | 3 |
| *FaRPa6Da* | 6D | 38.1 | Affx-88890456 | RLK, TMCC, NBS | **35.8** | 4 | Additive | 3 |
| *FaRPa7Aab* | 7A | 12.9 | **Affx-88892535** | RLP, TMCC | 24.1 | 2 | Additive | 3 |
| *FaRPa7Ba* | 7B | 12.0 | Affx-88892929 | TMCC | 12.6 | 1 | Dominant | 3 |
| *FaRPa7Ca* | 7C | 9.4 | Affx-88896002 | RLK | 10.6 | 1 | Hetero dominant | 3 |
| *FaRPa7Dab* | 7D | 19.8 | **Affx-88899847** | RLK, NBS | **36.9** | 5 | Additive | 3 |
|  |  |  |  |  |  |  |  |  |
| 2022 |  |  |  |  |  |  |  |  |
| **QTN name** | **Linkage group** | **Position (Mb)** | **Closest SNP** | **Type of Gene** | **Effect size (%)** | **Gene No** | **Model** | **Alleles** |
| *FaRPa1Ab* | 1A | 14.8 | Affx-88817415 | NBS, TMCC | 26.7 | 2 | Additive | 3 |
| *FaRPa1Bab* | 1B | 14.8 | **Affx-88817415** | NBS, TMCC | 26.7 | 2 | Additive | 3 |
| *FaRPa1Cb* | 1C | 8.8 | Affx-88902877 | RLK | 29.6 | 1 | Additive | 3 |
| *FaRPa3Ab* | 3A | 30.9 | Affx-88843644 | RLP, NBS, RPL | **48.1** | 8 | Additive | 3 |
| *FaRPa3Bb* | 3B | 9.5 | Affx-88843060 | MLO | 27.2 | 16 | no minor hom | 2 |
| *FaRPa3Dab* | 3D | 14.6 | **Affx-88838088** | RLK, RLP | **48.9** | 2 | no minor hom | 2 |
| *FaRPa4Bab* | 4B | 7.7 | **Affx-88848257** | TMCC | **38.2** | 1 | Additive | 3 |
| *FaRPa4Db* | 4D | 22.2 | Affx-88854014 | RLK | 27.7 | 1 | Dominant | 3 |
| *FaRPa5Aab* | 5A | 2.3 | **Affx-88859881** | TMCC, RLK | **31.9** | 3 | Additive | 3 |
| *FaRPa5Bb* | 5B | 15.0 | Affx-88866774 | RLK | **50.5** | 1 | no minor hom | 2 |
| *FaRPa5Cb* | 5C | 10.5 | Affx-88863794 | RLP | **50.4** | 1 | Additive | 3 |
| *FaRPa6Ab* | 6A | 33.7 | Affx-88888706 | NBS | **40.4** | 3 | Additive | 3 |
| *FaRPa6Bb* | 6B | 6.6 | Affx-88876401 | RLK, RLP | 22.6 | 4 | Dominant | 3 |
| *FaRPa6Cb* | 6C | 7.0 | Affx-88876085 | RLK, RLP | **78.7** | 4 | Additive | 3 |
| *FaRPa6Db* | 6D | 39.6 | Affx-88904022 | RLK | 24.2 | 3 | Additive | 3 |
| *FaRPa7Aab* | 7A | 12.9 | **Affx-88892535** | RLP, TMCC | 28 | 2 | Dominant | 3 |
| *FaRPa7Bb* | 7B | 15.4 | Affx-88897245 | RLK, NBS | 6.33 | 2 | Hetero recessive | 3 |
| *FaRPa7Cb* | 7C | 13.6 | Affx-88892283 | RLK | **61.1** | 1 | Additive | 3 |
| *FaRPa7Dab* | 7D | 19.8 | **Affx-88899847** | RLK, NBS | **60.5** | 5 | Additive | 3 |


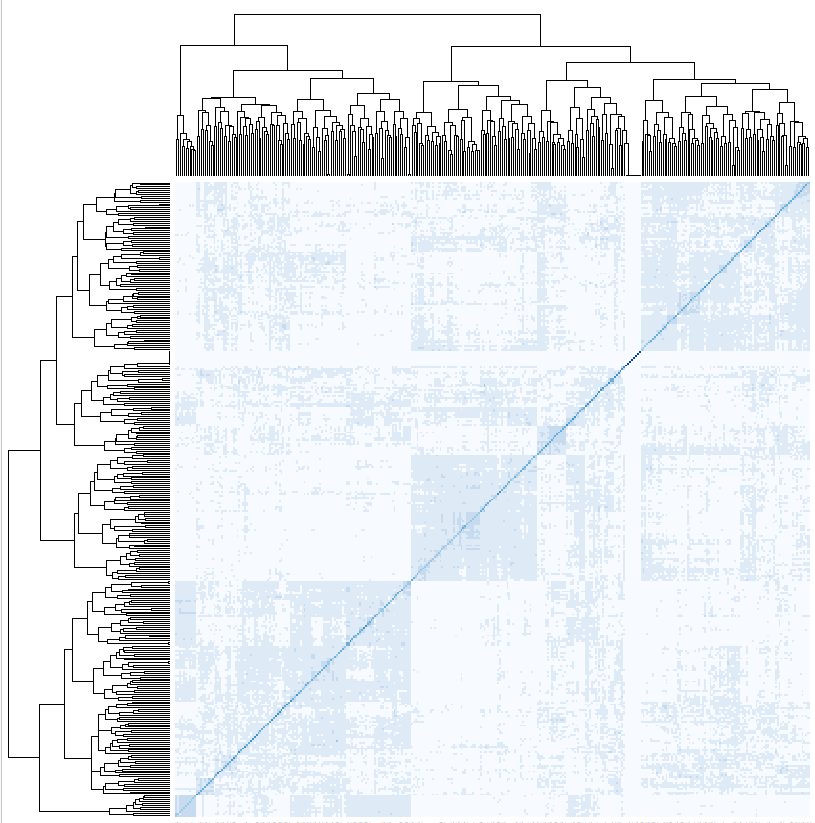
 **Supplementary Figure S1:** Genetic relationship matrix across 331 strawberry genotypes individuals used in the Genome Wide Association Study to detect powdery mildew resistance genes.


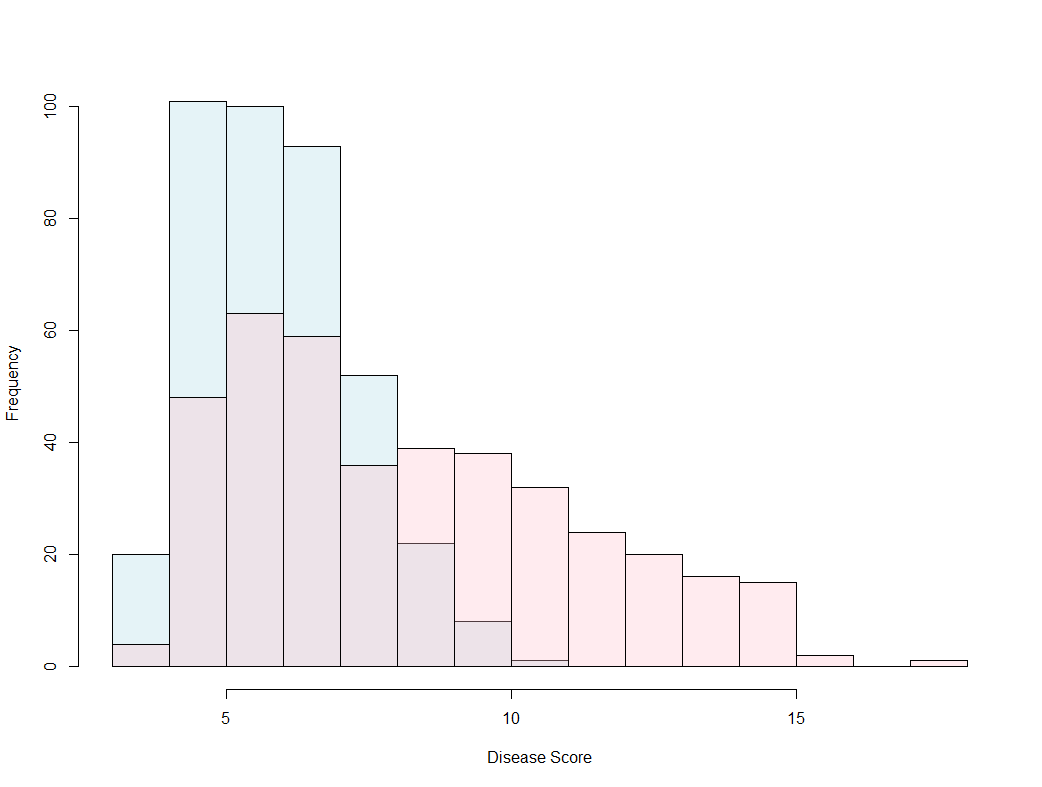


**Supplementary Figure S2:** Frequency histogram to show powdery mildew foliage disease scores in 2021 (Blue) and 2022 (Pink) for each genotype. Overlapping values (Purple) Values are Best Linear Unbiased Estimates for each genotype calculated from the Area Under the Disease Progression Curve scores that have been corrected for spatial variation.


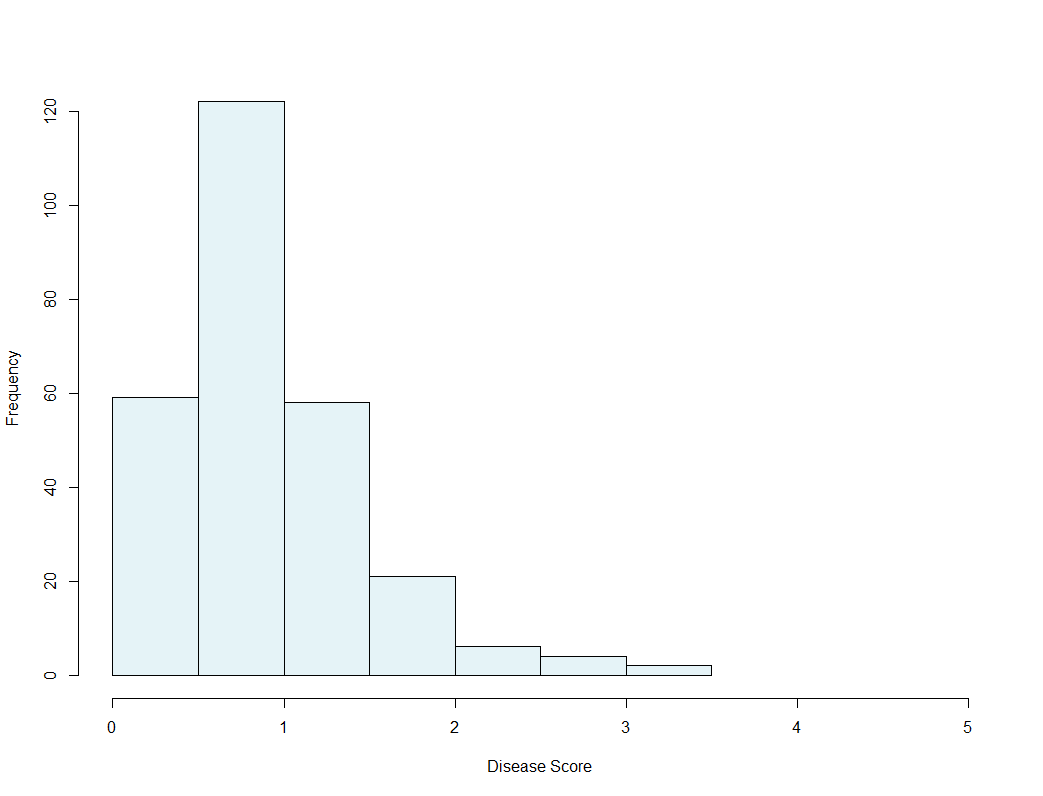


**Supplementary Figure S3:** Frequency histogram to show powdery mildew fruit disease scores in 2022 for each genotype. Values are Best Linear Unbiased Estimates for each genotype calculated from the weighted averages across pseudo-replicate berries that have been corrected for spatial variation.


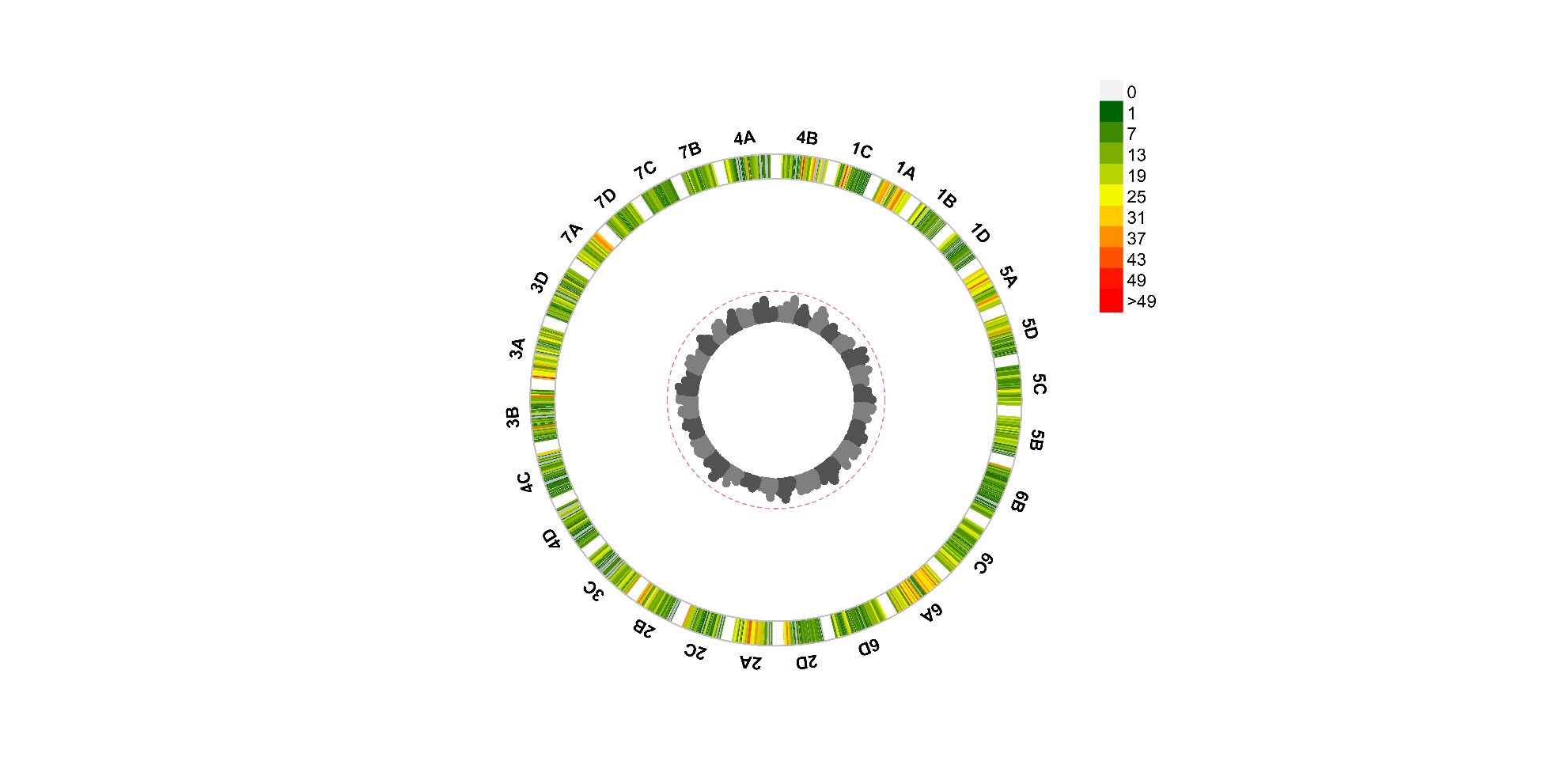


**Supplementary Figure S4:** A Manhattan plot of SNP markers across the 28 linkage groups of *Fragaria × ananassa* illustrating the relative association of SNPs with powdery mildew fruit disease symptom expression. Points represent markers. Blue points represent markers that fall above the-log10(*p*) significance threshold represented by the black dotted line. The inner circle represents the SNP associations with fruit powdery mildew disease symptoms. The outer circle represents the density of SNPs present on each chromosome within a 1 Mb window, the key represents the number of SNPs segregating from 0 to >49.
